# Health Care Access Barriers Bring Children to Emergency Rooms More Frequently: A Representative Survey

**DOI:** 10.1089/pop.2018.0089

**Published:** 2019-05-30

**Authors:** Thom Taylor, Daria Salyakina

**Affiliations:** Nicklaus Children's Research Institute, Miami, Florida.

**Keywords:** barriers, health care access, emergency department, children, caregivers

## Abstract

Children may visit the emergency department (ED) regularly in part because they and their caregivers may be experiencing barriers to appropriate and timely pediatric care. However, assessing the wide range of potential barriers to access to care that children and their caregivers may experience is often a challenge. The objective of this study was to assess the barriers to pediatric health care reported by caregivers and to examine the association between those reported barriers to care with the frequency of children's ED visits in the past 12 months. Assessment of ED utilization and access to care barriers was made through a telephone interview survey conducted as part of a broader Community Health Needs Assessment in 2015. A weighted community sample of adult caregivers (N = 1057) of children between the ages of 0–17 residing in Miami-Dade, Broward, and Palm Beach counties, Florida were contacted. This study found that multiple ED visits (≥2 vs. 0) in the past 12 months by a child were most strongly associated with access to care barriers attributed to language and culture (relative risk [RR] = 2.51), trouble finding a doctor (RR = 1.86), scheduling an appointment (RR = 1.68), and transportation access (RR = 1.73). These findings suggest that access to care barriers experienced by households may exacerbate the risk of a child experiencing repeated visits to the ED in a year. Findings are discussed further in the context of actionable population health management strategies to reduce risk of frequent ED utilization by children.

## Introduction

The emergency department (ED) is not always the optimal source of care for children,^1,2^ though the challenges and risk factors for repeated ED utilization that children and their caregivers face may be diverse and extensive.^3^ Considerations identified previously that affect the likelihood of frequent ED visits include whether a child has a chronic condition,^3^ younger age of the child,^3,4^ as well as socioeconomic status (SES) indicators.

Specifically, in the United States of America, poorer, unemployed individuals, nonimmigrants, and black or African Americans appear to have higher utilization of EDs.^4–6^ The source of health insurance for a child is also often linked to frequent ED utilization. Commonly, the child is less likely to utilize the ED if he or she has private insurance relative to public forms of insurance (eg, Medicaid).^7,8^ Inconsistent health coverage also may lead to repeated ED utilization as lack of health insurance can result in delayed care, unfilled prescriptions, limited well-child visits, and fractured health care for children.^9,10^

Even when a child has some form of health insurance, some caregivers still may struggle to find regular sources of care if fewer and fewer providers accept the child's health insurance. Such narrowing of networks, defined as a network that is “sufficient in number and type of providers”^11^ may make regular care for one's child more difficult.^10^ Recent evidence suggests that pediatric care may suffer from even more narrow networks than adult care.^12^

Moreover, even when a child is able to be seen by a provider in a particular network, attaining a timely appointment for regular care may be another hurdle.^1^ Still, when an appointment is available, office hours for the provider might conflict with other responsibilities of the caregiver.^8^ In tandem, the cost of care also may be a significant challenge for some caregivers.^12^ Additionally, should a child require medication, the cost of medication also may be prohibitive depending on income, co-pays, or the general expense of the medication the child may need.

Yet another challenge faced by some caregivers that does not always receive emphasis in health care is efficient transportation.^12,13^ For example, lack of a personal vehicle may force caregivers to rely on the schedules of relatives and peers for rides, or when available in an area, public transportation schedules that are potentially challenging. As a result, more limited access to efficient and reliable transportation may inadvertently make timely access to care for a child more difficult as well.^13^

Finally, cultural and language challenges are an important consideration in receiving appropriate care as communication between caregivers and community health care systems can be strained because of language or culturally-related communication barriers.^6^ This may manifest when recent immigrant populations attempt to navigate complex health care systems in a language unfamiliar to them.^14^ Yet, language and culture are not equivalent, and consideration of culture in the context of health care beliefs and expected practices of physicians also may be relevant when seeking health care.^15^

The aforementioned challenges and potential barriers to access to timely and appropriate care for children are not widely or comprehensively assessed in a single study. This study sought to consider the wide range of potential barriers to access to care that caregivers of children may face while simultaneously accounting for macro-level indicators of risk for ED utilization. The primary objective of this study was to assess the role that the wide range of aforementioned barriers to access to care may have on past 12-month ED utilization by a child.

## Methods

### Study design, setting, population, and sample

A weighted sample of residents in South Florida's Miami-Dade, Broward, and Palm Beach counties was surveyed on their landlines or cell phones by Professional Research Consultants Inc. (PRC) as part of a Child and Adolescent Community Health Needs Assessment (CHNA). For the telephone-administered survey, PRC assigned a caller ID number that is local to the area, made multiple attempts to reach respondents, and called at different times of day on different days of the week. The sample was stratified by county to ensure adequate representation among all strata.

At the time of the interview, for households with multiple children age 0–17, one child was selected at random based on which had the most recent birthday, and survey questions were asked about that specific child. This produced a sample that is more representative by demographics of age and sex. Survey respondents were adults ages 18 years and older who had at least 1 child residing in the household for whom the respondent was the health care decision maker. This person is referred to as the child's caregiver. Prior to completion of the telephone interview survey caregivers provided verbal consent to participate in the CHNA telephone survey interview.

Caregivers could respond in the 2 most dominant languages in South Florida communities: Spanish and English. PRC maintains a team of native Spanish-speaking interviewers who manage calls for those respondents who speak only Spanish or are more comfortable speaking Spanish. A parallel Spanish structured survey form conveying the same sentiments in Spanish as the English form also was used when conducting the interview with Spanish-speaking caregivers. In this way, the study team was able to ensure coverage of the language considerations for a very large proportion of the population in the counties involved.

Interviews were conducted throughout 2015 with a total of N = 1057 caregivers who completed the survey interview. Data were de-identified prior to investigators receiving the data and an Institutional Review Board exempt research protocol was approved for this study.

### Survey content and administration

Items in the CHNA survey inquired about health care access, health insurance, chronic conditions, and care utilization. Prior to de-identification, responses were weighted by PRC for nonresponse and further post-stratified based on sample characteristics of the child's age, sex, race/ethnicity, and household poverty status in order to align the survey responses to known population proportions using US Census Bureau population estimates.

The outcome of interest was the response to the following phone interview question: “In the past 12 months, how many times has the child gone to the hospital emergency room about (his/her) own health?” The study team was centrally interested in the question of which barriers may be associated with multiple visits to an ED in the past 12 months. Because of a very limited distribution beyond 2 visits in the past 12 months, each outcome was collapsed into 3-category multinomial responses with the levels *no visits*, *a single visit*, and *multiple (≥2) visits*. Health care access difficulty items assessed in the CHNA survey are presented in Table 1. All items were binary in response choice (*yes/no*).

**Table 1.** Community Health Needs Assessment Survey Items Addressing Access to Care Difficulties

1. Was there a time in the past 12 months when this child needed medical care, but you had difficulty finding a doctor?
2. Was there a time in the past 12 months when you had difficulty getting an appointment for this child to see a doctor?
3. Was there a time in the past 12 months when this child needed to see a doctor, but could not because of the cost?
4. Was there a time in the past 12 months when a lack of transportation made it difficult or prevented this child from seeing a doctor OR kept you from making a medical appointment for this child?
5. Was there a time in the past 12 months when this child was not able to see a doctor because the office hours were not convenient?
6. Was there a time in the past 12 months when this child needed a prescription medicine, but did not get it because you could not afford it?
7. Was there a time in the past 12 months when cultural or language differences made it difficult or prevented you from getting health care for this child?

### Data analysis

Limited item-level missing data (<4%) for any 1 variable was observed. Nevertheless, to ensure complete weighted sample estimation, random forest imputation methods^16^ were utilized to impute item-level nonresponse; the survey weight was incorporated in missing data imputation. Subsequently, a weighted multinomial model was fit with the focal variables of barriers to care described in Table 1. This model also adjusted for the child's age; sex; race/ethnicity combinations as captured in the CHNA including non-Hispanic white, non-Hispanic black, Hispanic, or other race/ethnicity combination; child having a chronic health condition; the household language; and the household poverty classification based on US Department of Health and Human Service (HHS) thresholds.^17^ Three HHS categories were assessed: household below 100% of the Federal poverty level, 100%-199% of the poverty level, and 200% and above the poverty level. Health insurance status of the child at the time of the phone interview (private, Medicaid, Medicare, other, or uninsured) was captured, as well as inconsistency in health care coverage.

Inconsistency in health care coverage was defined as any period in the child's life when he or she did not have health insurance. County of residence also was accounted for given that Miami-Dade, Broward, and Palm Beach counties vary demographically and socioeconomically in a manner similar to other large metropolitan areas in the United States.

The multinomial model also incorporated sampling and nonresponse weights to provide population-level inference. Relative risk (RR) estimates and associated 95% confidence intervals (CIs) were estimated as were the average adjusted marginal probabilities and associated 95% CIs from the predicted model estimates.

## Results

First, the weighted distribution of ED visit frequency in the sample and sample demographics were evaluated. The majority of the children in this study (66%) had not visited the ED in the past 12 months. Approximately 18% of caregivers reported that their child had visited the ED once, and a still sizeable 16% had ≥2 ED visits in the past 12 months (Table 2). Detailed weighted descriptive estimates of ED visits, demographics, and access barriers are presented in the following text and in Table 2.

**Table 2.** Demographics and Outcomes

*Variable*	*Level*	*Weighted n*	*Weighted percent (95% CI)*
Outcomes			
Emergency Department Visits in Past 12 Months	0 Visits	694	66% (62%–69%)
	1 Visit	195	18% (16%–21%)
	≥2 Visits	169	16% (13%–19%)
Urgent Care Visits in Past 12 Months	0 Visits	669	63% (60%–67%)
	1 Visit	227	21% (19%–24%)
	≥2 Visits	162	15% (13%–18%)
**Demographics**			
Sex of Child	a. Male	541	51% (48%–55%)
	b. Female	517	49% (45%–52%)
Race/Ethnicity of Child	a. Hispanic	446	42% (39%–45%)
	b. NHW	287	27% (25%–30%)
	c. NHB	254	24% (21%–27%)
	d. Other	72	7% (5%–9%)
Child has Chronic Condition	a. No	856	81% (78%–84%)
	b. Yes	202	19% (16%–22%)
Insurance Type Child has	a. Private Health Insurance	497	47% (44%–50%)
	b. Medicaid	306	29% (25%–32%)
	c. Medicare	91	9% (6%–11%)
	d. Other	79	8% (6%–9%)
	e. Uninsured	85	8% (6%–10%)
Child has had Inconsistent Health Insurance Coverage	a. No	920	87% (84%–89%)
	b. Yes	138	13% (11%–16%)
Household HHS Poverty Classification	a. 200% FPL or Higher	601	57% (53%–60%)
	b. 100% to 199% of FPL	239	23% (20%–25%)
	c. Below FPL	219	21% (17%–24%)
Household Language	a. English	856	81% (78%–84%)
	b. Spanish	141	13% (11%–16%)
	c. Other or Mix	61	6% (4%–7%)
County of Residence	a. Miami-Dade	479	45% (44%–47%)
	b. Broward	343	32% (31%–34%)
	c. Palm Beach	236	22% (21%–24%)
**Access to Care Difficulties**			
1. Access difficulty finding a doctor	a. No	920	87% (84%–89%)
	b. Yes	138	13% (11%–16%)
2. Access difficulty getting appointment	a. No	852	81% (78%–83%)
	b. Yes	206	19% (17%–22%)
3. Access difficulty due to cost of doctor	a. No	916	87% (84%–89%)
	b. Yes	142	13% (11%–16%)
4. Access difficulty due transportation to doctor	a. No	952	90% (88%–92%)
	b. Yes	106	10% (8%–12%)
5. Access difficulty due to available office hours	a. No	860	81% (78%–84%)
	b. Yes	198	19% (16%–22%)
6. Access difficulty due Rx cost	a. No	943	89% (87%–91%)
	b. Yes	116	11% (9%–13%)
7. Access difficulty due to cultural or language differences	a. No	996	94% (92%–96%)
	b. Yes	62	6% (4%–8%)

Note: Weighted estimates may not always sum to the total sample size of N = 1057 because of rounding error of the weighted estimates.

CI, confidence interval; FPL, Federal poverty level; HHS, Department of Health and Human Services; NHB, non-Hispanic black; NHW, non-Hispanic white; Rx, prescription.

### Patient demographics and insurance type

**FIG. 1.**
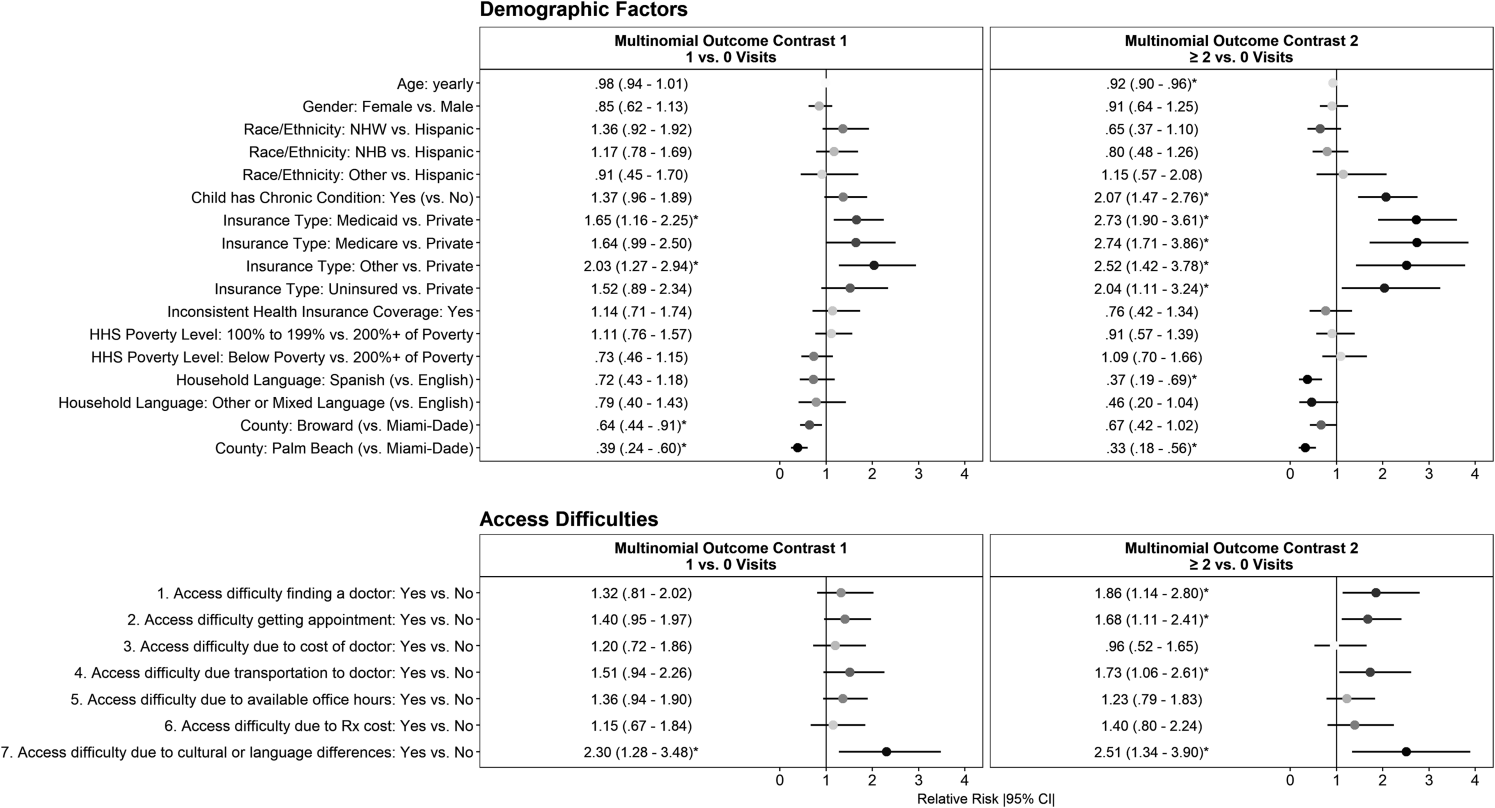
Relative risk estimates to visit the emergency department ≥1 time(s) by a child associated with demographic factors and access to care barriers. All relative risk estimates with 95% confidence interval *not* crossing the *vertical black lines* (marked with “*”) are statistically significant at *P* < .05. HHS, US Department of Health and Human Services; NHB, non-Hispanic black; NHW, non-Hispanic white.

**FIG. 2.**
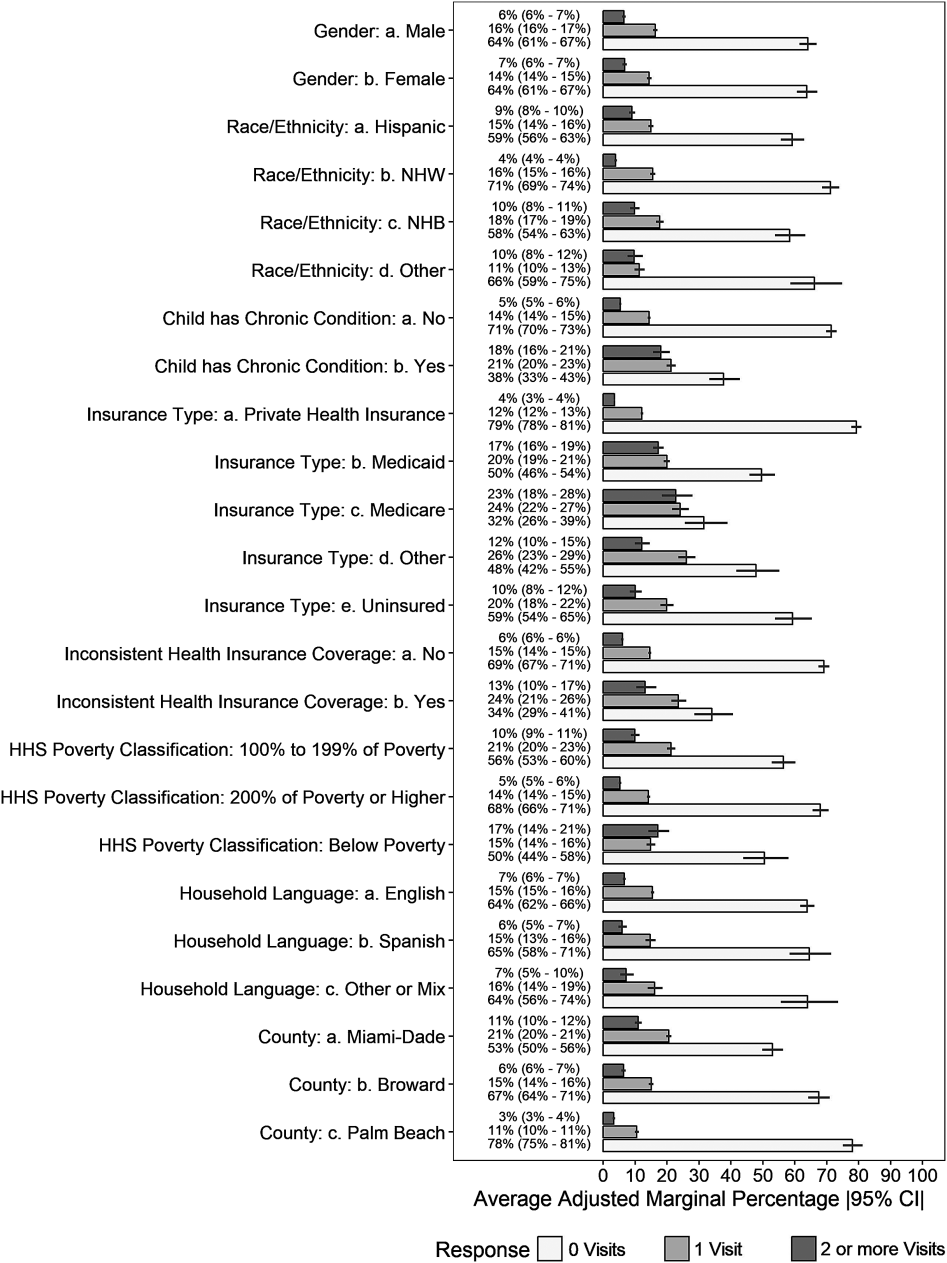
Probabilities of emergency department visit frequencies by different demographic subgroups. Probabilities are presented as weighted multivariate adjusted average marginal predicted percentages for each demographic covariate included in the multinomial model predicting multiple visits (≥2), a single visit (1), or no (0) visits in the past 12 months. Each bar represents the average marginal predicted point estimate for each level of the covariate by 3 categories of the emergency department visit frequency. *Black horizontal lines* represent the 95% confidence interval (CI) of the estimate. HHS, US Department of Health and Human Services; NHB, non-Hispanic black; NHW, non-Hispanic white

Figure 1 presents the RR estimates and Figure 2 presents the corresponding average marginal probabilities for the associated RR estimates of the adjusted multinomial model. Age of the child at the time of the interview was only associated with ≥2 vs. no ED visits in the past 12 months (RR = .92, *P* < .05) (Figure 1). Neither sex nor race/ethnicity was significantly associated with frequency of ED visits.

Unsurprisingly, children with a chronic condition did have a higher risk of multiple ED visits, (RR = 2.07, *P* < .05). Yet, no association between ED visits and certain socioeconomic considerations was observed (ie, poverty status). However, Spanish language use in the home was associated with a reduced likelihood of multiple ED visits (RR = .37, *P* < .05). Additionally, county of residence was associated with both single and multiple visits to the ED. Relative to residents of Miami-Dade County, children in Broward and Palm Beach were less likely to have a single ED visit (RR = .64, *P* < .05 and RR = .39, *P* < .05, respectively). A similar pattern was observed for children reported to have had multiple ED visits in the past 12 months (RR = .67, *P* < .05 and RR = .33, *P* < .05, respectively).

Relative to children having private insurance, having Medicaid insurance was associated with multiple ED visits (RR = 2.73, *P* < .05) in the past 12 months (Figure 1). Similarly, children with Medicare had a higher risk of ≥2 visits (RR = 2.74, *P* < .05) (Figures 1 and 2). Children who did not have insurance also were twice as likely to have been in the ED multiple (≥2) times relative to privately insured children (RR = 2.04, *P* < .05). Similar effects were found with none vs. a single visit, though variability in those estimates led to less confidence about the robustness of these findings at the population level.

### Access to care barriers

**FIG. 3.**
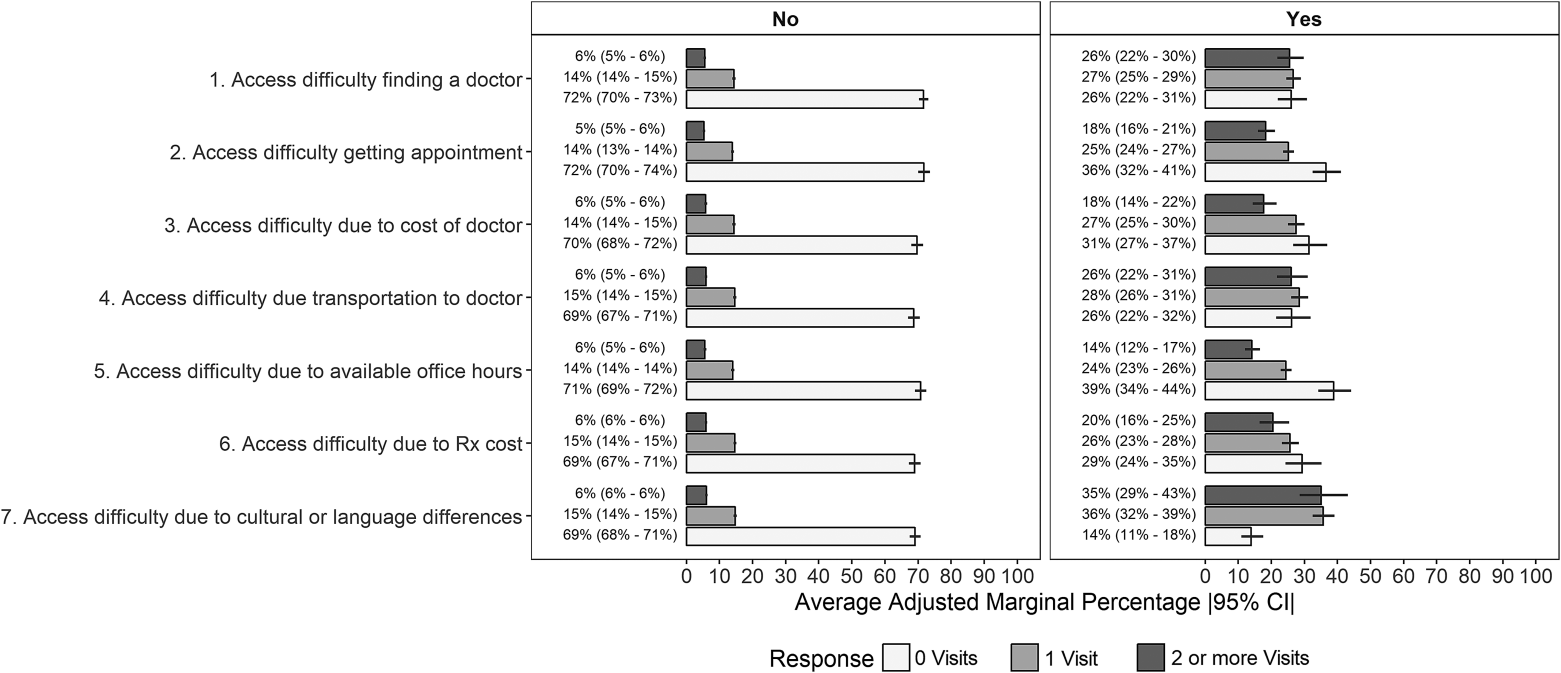
Probabilities of emergency department visit frequencies by different access to care barriers experienced by caregivers. Probabilities are presented as weighted multivariate adjusted average marginal predicted percentages for each pediatric health care access difficulty included in the multinomial model predicting a child's emergency department (ED) visits in the past 12 months. The *left-hand panel* presents the adjusted percentages for caregivers who reported they *did not* experience the respective access difficulty listed on the ordinate in the past 12 months. The *right-hand panel* presents the adjusted percentages for caregivers who reported they *did* experience the respective access difficulty listed on the ordinate in the past 12 months. Each bar represents the average marginal predicted point estimate for each level of the covariate by each category of the ED visit. *Black horizontal lines* represent the 95% confidence interval (CI) of the estimate.

Out of 7 assessed barriers to health care reported by children's caregivers in the survey, 4 were statistically significantly associated with frequency of multiple ED visits. The most prominent and consistent association was found for cultural or language differences. Overall, this was not a common barrier as it was reported by only an estimated 6% (95% CI: 4%–8%) of caregivers (Table 2). Yet, it was associated with both a single (RR = 2.30, *P* < .05) and multiple ED visits (RR = 2.51, *P* < .05) in the past 12 months (Figure 2). Caregivers who reported experiencing cultural or language barriers to care were roughly 5.8 times more likely to report their child had ≥2 ED visits (35% vs. 6%) and 2.5 times more likely to report a single ED visit (36% vs. 15%) with the child within the last 12 months when compared to caregivers who did not report experiencing this difficulty (Figure 3).

Further, difficulty finding a doctor for the child, difficulty making an appointment, and reported transportation challenges were associated with multiple ED visits (RR = 1.86, *P* < .05; RR = 1.68, *P* < .05; RR = 1.73, *P* < .05, respectively), but not with single ED visits (Figures 2 and 3). Those caregivers who reported that they had difficulty finding a doctor for their child in the past 12 months were 4.3 times more likely to bring their child to the ED ≥2 times (26% vs. 6%). Similarly, caregivers who experienced difficulty making an appointment or transportation challenges were 3.6 (18% vs. 5%) and 4.3 (26% vs 6%) times more likely to bring their child to the ED multiple times in a 12-month period (Figure 3). The cost of a doctor, inconvenience of office hours, and the cost of medications were not associated with past 12-months ED visit frequency.

## Discussion

Of central interest to the objectives of this study, the findings support the notion that demographics, comorbidities, *and* access to care barriers experienced in the household by caregivers may all affect ED utilization in a 12-month period. These findings highlight that caregivers who experienced barriers to appropriate access to health care for their children often may rely on the ED as a primary source of such care for their children.^5^

At the same time, the findings align well with previous research assessing population health dynamics and care utilization rates at a national level. For example, the Children's Hospital Association reported that upward of 37% of children in care in pediatric hospitals may be admitted to an ED at least once in a year.^3^ Results from the present population-weighted survey converge with that estimate. More notably, the present study found 16% or more of children potentially visiting the ED multiple times in a year.

Although the present study found a consistent relationship between younger age of the child and greater ED utilization, common demographic and SES associations with pediatric ED visits encountered in the pediatric literature^3–5,18^ were not entirely consistent with these results. Specifically, there was a relatively small influence of ethnicity and poverty status on ED utilization. To some degree, this may reflect the limited information carried by the broad categorizations used in the survey. For instance, categorizations of a race and Hispanic ethnicity may wash away meaningful variability among different Hispanic groups residing in South Florida. Hispanic or Latino residents in South Florida come from a diverse range of populations comprising very different socioeconomically stratified countries of origin.^19^ Additionally, the 3-category HHS poverty status metric available to this study may have been relatively coarse in categorization of potential economic hardship. Nonetheless, this study was able to analyze a number of access to care barriers experienced by more disadvantaged groups that usually are not measured in other data sources.

At a macro level, the findings in this study further highlight the significance of access barriers related to SES. Although Miami-Dade, Broward, and Palm Beach are contiguous, Census estimates show that different subpopulations reside in each of these counties. In particular, Miami-Dade county has more recent immigrants from the Caribbean, and Central and South America than Broward or Palm Beach counties.^19,20^ Moreover, relative to the United States as a whole as well as Broward and Palm Beach counties, Miami-Dade county households are poorer, are living in poverty, have lower levels of educational attainment, have less health insurance coverage, have more family members living in the same residence, and also face issues associated with more dense urban environments such as longer wait times in transit.^19,20^

Present study findings converge on a general consideration that largely immigrant communities who may not speak English may face multiple barriers to access to care. In addition, immigration status also may play a relevant role in seeking care before an emergency arises. Specifically, evidence suggests that bureaucratic and registration systems may hinder access to timely care for undocumented immigrants, as does a general lack of familiarity with US health care systems.^21^

With respect to the language spoken in the household and similar acculturation dynamics, households that are less US acculturated tend to have a lower likelihood of ED visits in a given time period relative to more acculturated households. As families acculturate to US norms, their ED utilization increases.^22^ One interpretation for this pattern may be that caregivers who are less acculturated may be less inclined to seek care anywhere, while those who are more acculturated find the ED a convenient place to seek care.^23^

At the same time, perceived convenience of the ED for care may indirectly hint at a general lack of health literacy that makes it difficult to navigate local health care systems. To the extent this interpretation is accurate, it offers an opportunity for health care-related educational interventions.^24^ Such educationally-oriented interventions can occur at the point of contact in the ED (ie, health care professionals) to better support patients who are likely to have primary care and specialty care follow-up needs.

Basic considerations regarding enrollment in a health insurance program may represent an opportunity for intervention as well. Consistent with much of the prior literature in both adult^25^ and pediatric care,^7,8,26^ having any form of public insurance was associated with an increased reliance on the ED. In part, this may reflect some degree of selection within which children with more medically or economically complex circumstances become eligible for public insurance options such as Medicaid, Medicare, and the Children's Health Insurance Program. Yet, some evidence suggests that those who have recently obtained health insurance coverage for themselves and their children still may retain some degree of low health literacy.^27^ As such, bolstering point-of-enrollment health care education may be beneficial to the caregiver, the health care institutions that may provide care to the child, and the public insurance system.

There also may be opportunities for intervention to decrease regular reliance on the ED by caregivers once an initial ED visit has occurred. For example, it is recommended that health care systems work to bolster their social work and case management support staff presence in the ED so that follow-up, education, and needs can be addressed more adequately at the point of care.^28,29^ This also may limit the time burdens faced by health care professionals when meeting with pediatric patients and their caregivers.

With respect to community support outside of the ED or hospital setting, one approach to improving care for children with chronic conditions gaining traction in recent years is an increased emphasis on promoting community health workers to support caregivers.^30^

The present study found that, among the access to care difficulties, cultural or language differences were most strongly related to multiple ED visits. Much evidence supports that ED utilization may increase when there is limited ability to communicate effectively with health care providers.^31,32^ Unfortunately, pediatric primary care may not always accommodate the language and cultural considerations of caregivers or children.^33^ Examples of basic components of culturally and language-conscious care include adequately listening to an individual, adequately answering questions, adequately explaining medications and their effects, and providing adequate empathy for the patient's situation.^34^ Doing this for both a young child and an adult may present extra challenges for providers in pediatric settings—particularly when there is a mismatch in language or health beliefs and perceptions.

In this respect, this study highlights what likely remains as an extra challenge faced by care providers in pediatric settings. Nevertheless, educational interventions at the point of care may represent opportunities for education on essential care follow-up between the provider and the child's caregiver(s).^24,35^ At the same time, this approach is not without significant challenges. For example, educational efforts and teach-back can be a challenge to effectively standardize^24^ and this speaks to the heterogeneity of health literacy needs that many caregivers may have—particularly in areas where diverse populations reside.

The potential role of narrow health care insurance networks and pediatric ED admissions also cannot be overlooked. The study team found that reports of difficulty finding a doctor for the child in the past 12 months were associated with an increased risk of multiple ED visits.

There is clear evidence that lack of available primary or specialty care for children in need tends to be associated with emergency care seeking^36^ and that improving access to primary care likely will diminish this behavior.^37^ Parallel with the importance of access to pediatric physicians overall, access to regular and convenient appointments for the child also were associated with ED visits in the past 12 months in the current study. To some degree, this may reflect a general narrowing of networks of available pediatric physicians who can meet the demand for care by the community in a timely fashion. Indeed, a mix of increased access to health insurance and a general narrowing of networks might cause demand for children's health care to outstrip available supply.^38^

Finally, this study found that access to care barriers attributed to transportation challenges also were associated with multiple ED visits. Here, the literature is somewhat mixed. On one hand, closer proximity to an ED is associated with a greater number of ED visits.^3^ On the other hand, lack of efficient and reliable transportation issues faced by many caregivers impacts their ability to bring a child to necessary care in a timely fashion.^13,39^ From this standpoint, health care systems could work to bolster the efficiency of other forms of care for children, such as more robust telehealth initiatives,^8,40^ that accommodate the language and technology device preferences of patients and caregivers.

### Limitations

Although the data in this study were weighted to reflect survey nonresponse and the demographically diverse population of urban South Florida, sampling did not occur in more rural areas of South Florida. As a result, generalizability to more rural communities may be limited. It also must be acknowledged that the wording of the item in the CHNA phone interview pertaining to language and culture was inadvertently asked as a “double-barreled” question (ie, 2 questions asked as 1); this may have introduced ambiguity in caregiver interpretation of that survey question. In the present study it remains difficult to disentangle the specific causal mechanisms resulting in ED visits that were found embedded within cultural and language barriers that caregivers may have experienced when seeking health care for the child.

## Conclusions

This representative survey found health care access barriers were associated with multiple pediatric ED visits in a 12-month period even after accounting for type of health insurance, poverty status, household language, child age, and race/ethnicity. Access barriers related to cultural and language challenges in particular may affect some caregivers' ability to seek timely care for their children. Difficulty finding a doctor and difficulty making an appointment were associated with multiple ED admissions in a year. This may reflect potential impacts of narrow networks on pediatric access to care. Additionally, caregivers may experience limited accessibility to convenient and accommodating pediatric primary care.

This could lead to an exacerbation of complications for some pediatric patients and perhaps also lead some caregivers to see the ED as the only viable option for care under their respective circumstances. However, access to care barriers measured in this study are potentially modifiable and appropriate interventions can lead to limiting unnecessary ED visits and the associated strain this places on children, caregivers, payers, and health care systems in general.

## References

[1] Farion KJ, Wright M, Zemek R, et al. Understanding low-acuity visits to the pediatric emergency department. PLoS One 2015;10:e0128927 26083338 10.1371/journal.pone.0128927 PMC4471269

[2] Kubicek K, Liu D, Beaudin C, et al. A profile of non-urgent emergency department usage in an urban pediatric hospital. Pediatr Emerg Care 2012;28:977–984 23023463 10.1097/PEC.0b013e31826c9aab PMC3464348

[3] Neuman MI, Alpern ER, Hall M, et al. Characteristics of recurrent utilization in pediatric emergency departments. Pediatrics 2014;134:e1025–e1031 25225134 10.1542/peds.2014-1362

[4] Zimmer KP, Walker A, Minkovitz CS Epidemiology of pediatric emergency department use at an urban medical center. Pediatr Emerg Care 2005;21:84–89 15699815 10.1097/01.pec.0000159050.19188.23

[5] Alpern ER, Clark AE, Alessandrini EA, et al. Recurrent and high-frequency use of the emergency department by pediatric patients. Acad Emerg Med 2014;21:365–373 24730398 10.1111/acem.12347

[6] Weech-Maldonado R, Morales LS, Spritzer K, Elliott M, Hays RD Racial and ethnic differences in parents' assessments of pediatric care in Medicaid managed care. Health Serv Res 2001;36:575–594 11482590 PMC1089243

[7] Nikpay S, Freedman S, Levy H, Buchmueller T Effect of the Affordable Care Act Medicaid expansion on emergency department visits: evidence from state-level emergency department databases. Ann Emerg Med 2017;70:215–225.e6 28641909 10.1016/j.annemergmed.2017.03.023

[8] Phelps K, Taylor C, Kimmel S, Nagel R, Klein W, Puczynski S Factors associated with emergency department utilization for nonurgent pediatric problems. Arch Fam Med 2000;9:1086 11115212 10.1001/archfami.9.10.1086

[9] Olson LM, Tang SS, Newacheck PW Children in the United States with discontinuous health insurance coverage. N Engl J Med 2005;353:382–391 16049210 10.1056/NEJMsa043878

[10] Cheng TL, Wise PH, Halfon N Promise and perils of the Affordable Care Act for children. JAMA 2014;311:1733–1734 24794362 10.1001/jama.2014.930 PMC4575119

[11] Grace AM, Horn I, Hall R, Cheng TL Children, families, and disparities: pediatric provisions in the Affordable Care Act. Pediatr Clin North Am 2015;62:1297–1311 26318953 10.1016/j.pcl.2015.06.003 PMC4826597

[12] Wong CA, Kan K, Cidav Z, Nathenson R, Polsky D. Pediatric and adult physician networks in Affordable Care Act marketplace plans. Pediatrics 2017 [Epub ahead of print]; DOI: 10.1542/peds.2016-3117 28250022

[13] Syed ST, Gerber BS, Sharp LK Traveling towards disease: transportation barriers to health care access. J Community Health 2013;38:976–993 23543372 10.1007/s10900-013-9681-1 PMC4265215

[14] Hampers LC, Cha S, Gutglass DJ, Binns HJ, Krug SE Language barriers and resource utilization in a pediatric emergency department. Pediatrics 1999;103(6 pt 1):1253–1256 10353938 10.1542/peds.103.6.1253

[15] Flores G. Culture and the patient-physician relationship: achieving cultural competency in health care. J Pediatr 2000;136:14–23 10636968 10.1016/s0022-3476(00)90043-x

[16] Stekhoven DJ, Bühlmann P MissForest—non-parametric missing value imputation for mixed-type data. Bioinformatics 2012;28:112–118 22039212 10.1093/bioinformatics/btr597

[17] US Department of Health and Human Services. 2015 Poverty Guidelines. 2015 https://aspe.hhs.gov/2015-poverty-guidelines Accessed 121, 2017

[18] Dy CJ, Lyman S, Do HT, Fabricant PD, Marx RG, Green DW Socioeconomic factors are associated with frequency of repeat emergency department visits for pediatric closed fractures. J Pediatr Orthop 2014;34:548–551 24590328 10.1097/BPO.0000000000000143 PMC4051828

[19] Motel S, Patten E. The 10 Largest Hispanic Origin Groups: Characteristics, Rankings, Top Counties. www.pewhispanic.org/files/2012/06/The-10-Largest-Hispanic-Origin-Groups.pdf Accessed 730, 2018

[20] US Census Bureau. QuickFacts Palm Beach County, Florida; Broward County, Florida; Miami-Dade County, Florida; UNITED STATES. 2018 www.census.gov/quickfacts/fact/table/US/PST045217 Accessed 730, 2018

[21] Hacker K, Anies M, Folb BL, Zallman L Barriers to health care for undocumented immigrants: a literature review. Risk Manag Healthc Policy 2015;8:175–183 26586971 10.2147/RMHP.S70173 PMC4634824

[22] Allen L, Cummings J Emergency department use among Hispanic adults: the role of acculturation. Med Care 2016;54:449–456 26908087 10.1097/MLR.0000000000000511 PMC4833554

[23] Nokoff N, Brunner AM, Linakis JG, Amanullah S Presentation to either the pediatric emergency department or primary care clinic for acute illness: the caregivers' perspective. Pediatr Emerg Care 2014;30:146–150 24583577 10.1097/PEC.0000000000000082

[24] Morgan SR, Chang AM, Alqatari M, Pines JM Non-emergency department interventions to reduce ED utilization: a systematic review. Acad Emerg Med 2013;20:969–985 24127700 10.1111/acem.12219 PMC4038086

[25] Zuckerman S, Shen Y-C Characteristics of occasional and frequent emergency department users: do insurance coverage and access to care matter? Med Care 2004;42:176–182 14734955 10.1097/01.mlr.0000108747.51198.41

[26] Stone ML, LaPar DJ, Mulloy DP, et al. Primary payer status is significantly associated with postoperative mortality, morbidity, and hospital resource utilization in pediatric surgical patients within the United States. J Pediatr Surg 2013;48:81–87 23331797 10.1016/j.jpedsurg.2012.10.021 PMC3921619

[27] Morrison AK, Myrvik MP, Brousseau DC, Hoffmann RG, Stanley RM The relationship between parent health literacy and pediatric emergency department utilization: a systematic review. Acad Pediatr 2013;13:421–429 23680294 10.1016/j.acap.2013.03.001 PMC3808118

[28] Kumar GS, Klein R Effectiveness of case management strategies in reducing emergency department visits in frequent user patient populations: a systematic review. J Emerg Med 2013;44:717–729 23200765 10.1016/j.jemermed.2012.08.035

[29] Van den Heede K, Van de Voorde C Interventions to reduce emergency department utilisation: a review of reviews. Health Policy Amst Neth 2016;120:1337–1349 10.1016/j.healthpol.2016.10.002 27855964

[30] Campbell JD, Brooks M, Hosokawa P, Robinson J, Song L, Krieger J Community health worker home visits for Medicaid-enrolled children with asthma: effects on asthma outcomes and costs. Am J Public Health 2015;105:2366–2372 26270287 10.2105/AJPH.2015.302685 PMC4605150

[31] Yu SM, Huang ZJ, Schwalberg RH, Nyman RM Parental English proficiency and children's health services access. Am J Public Health 2006;96:1449–1455 16809589 10.2105/AJPH.2005.069500 PMC1522110

[32] Rogers AJ, Delgado CA, Simon HK The effect of limited English proficiency on admission rates from a pediatric ED: stratification by triage acuity. Am J Emerg Med 2004;22:534–536 15666256 10.1016/j.ajem.2004.08.012

[33] Seid M, Stevens GD, Varni JW Parents' perceptions of pediatric primary care quality: effects of race/ethnicity, language, and access. Health Serv Res 2003;38:1009–1032 12968814 10.1111/1475-6773.00160 PMC1360930

[34] Morales LS, Cunningham WE, Brown JA, Liu H, Hays RD Are Latinos less satisfied with communication by health care providers? J Gen Intern Med 1999;14:409–417 10417598 10.1046/j.1525-1497.1999.06198.x PMC1496614

[35] Sturm JJ, Hirsh D, Weselman B, Simon HK Reconnecting patients with their primary care provider: an intervention for reducing nonurgent pediatric emergency department visits. Clin Pediatr (Phila) 2014;53:988–994 25006110 10.1177/0009922814540987

[36] Stingone JA, Claudio L Disparities in the use of urgent health care services among asthmatic children. Ann Allergy Asthma Immunol 2006;97:244–250 16937759 10.1016/S1081-1206(10)60021-X

[37] Cecil E, Bottle A, Cowling TE, Majeed A, Wolfe I, Saxena S Primary care access, emergency department visits, and unplanned short hospitalizations in the UK. Pediatrics 2016;137:e20151492 26791971 10.1542/peds.2015-1492

[38] Institute of Medicine. Transforming Health Care Scheduling and Access: Getting to Now. Washington, DC: National Academies Press, 2015. 26378331

[39] Nance ML, Carr BG, Branas CC Access to pediatric trauma care in the United States. Arch Pediatr Adolesc Med 2009;163:512–518 19487606 10.1001/archpediatrics.2009.65

[40] Keely E, Liddy C, Afkham A Utilization, benefits, and impact of an e-consultation service across diverse specialties and primary care providers. Telemed J E Health 2013;19:733–738 23980939 10.1089/tmj.2013.0007 PMC3787335

